# NLRP3 inflammasome activation By 17β-estradiol is a potential therapeutic target in hepatocellular carcinoma treatment

**DOI:** 10.1007/s12032-022-01945-z

**Published:** 2023-02-10

**Authors:** Sara F. Awwad, Raymonde H. Assaf, Ahmed A. Emam, Amgad A. Fouad, Lamiaa F. Arafa, Aya A. El-Hanafy

**Affiliations:** 1 Department of Medical Biochemistry and Molecular Biology, Faculty of Medicine, Mansoura University, Mansoura, Egypt; 2 Gastroenterology Surgical Center, Faculty of Medicine, Mansoura University, Mansoura, Egypt; 3 Medical Experimental Research Center (MERC), Faculty of Medicine, Mansoura University, Mansoura, Egypt

**Keywords:** Hepatocellular carcinoma, NLRP3 inflammasome, Gasdermin-D, Pyroptosis, 17β-estradiol

## Abstract

Hepatocellular carcinoma (HCC) is one of the most common cancers worldwide, and it mostly arises as a consequence of persistent chronic inflammation. Recently, NLRP3 inflammasome has caught the attention of many research groups due to its involvement in different types of cancer. However, its direct role in HCC remains elusive. Our study aimed to evaluate the role of NLRP3 inflammasome and pyroptosis in HCC and to clarify the potential mechanism by which 17β-estradiol (E2) can be used as a protective factor against HCC. NLRP3, caspase-1 (CASP1) as well as gasdermin-D (GSDMD) mRNA expression levels were assessed in human HCC tissues and adjacent non-cancerous liver tissues. Also, HepG2 HCC cells were cultured and treated with E2, followed by detection of the mRNA levels of these three genes. Our results revealed that NLRP3, CASP1, and GSDMD mRNA expressions were significantly lower in HCC tissues than in controls, and this under-expression was closely correlated with advanced HCC stages and grades. In contrast, HepG2 HCC cells displayed significantly higher expression levels of NLRP3 inflammasome components and GSDMD in the two E2-treated groups compared to the untreated group. Also, NLRP3, CASP1, and GSDMD mRNA expression levels were positively correlated with each other. This study confirmed that lack of NLRP3 inflammasome is involved in HCC progression and 17β-estradiol-induced activation of NLRP3 inflammasome may be effective in HCC treatment as it inhibited tumor cell growth and proliferation by triggering CASP1-dependent pyroptosis in HCC cells.

## Introduction

Hepatocellular carcinoma represents the most common malignancy of primary liver cancers and the third leading cause of cancer-related death worldwide [1]. In Egypt, HCC is considered a major public health problem due to the high prevalence of hepatitis C viral (HCV) infection [2]. It mostly occurs in an established background of chronic liver disease especially cirrhosis which may be caused by persistent HCV or hepatitis B viral (HBV) infection, non-alcoholic fatty liver disease/non-alcoholic steatohepatitis (NAFLD/NASH), or alcoholic liver disease (ALD) [3].

One key step in the inflammatory response, generated by the innate immunity to protect the liver against various pathogens and danger signals, is the activation of cytosolic multiprotein complexes known as inflammasomes. However, excessive inflammatory responses regulated by these complexes can trigger the progression of various liver diseases [4]. Several inflammasomes have been described. Among them, “the nucleotide-binding oligomerization domain-like receptor-pyrin domain containing protein 3 (NLRP3) inflammasome” is the most characterized one and its activation mechanism has been widely investigated. It consists of three components; (1) sensor; (NLRP3), (2) adaptor; apoptosis-associated speck-like protein containing a caspase activation and recruitment domain (ASC), and (3) effector protease; (pro-caspase-1) [5]. Upon activation, these three components are assembled to process the autocatalytic cleavage of pro-caspase-1 and its activation to caspase-1 (active form) which consequently cleaves the pro-inflammatory cytokines (interleukin-1β (IL-1β) and IL-18) allowing their maturation, secretion and finally resulting in inflammation. Also, caspase-1 can activate a cytosolic protein known as gasdermin-D (GSDMD) which is an executioner of pyroptosis [6].

The NLRP3 inflammasome was demonstrated to promote the pathogenesis and progression of various liver diseases such as viral hepatitis, NASH, hepatic fibrosis, cirrhosis, and HCC [7]. Its components are expressed in Kupffer cells (KCs), liver sinusoidal endothelial cells (LSECs), hepatic stellate cells (HSCs), and periportal myofibroblasts participating directly or indirectly in the promotion of liver fibrosis. The NLRP3 inflammasome is considered to be a double-edged sword in cancer as it may either promote or suppress tumor growth. However, its direct role in liver cancer remains poorly described and elusive [8].

Pyroptosis is a pro-inflammatory type of programmed cell death in which the cells can identify certain danger signals, produce cytokines, swell, burst, release the intracellular pro-inflammatory contents and finally die [9]. Pyroptosis has been demonstrated to be associated with various diseases including cardiovascular, metabolic, and immune-related diseases and cancers. For cancer cells, the induction of pyroptosis was found to either produce beneficial effects against many cancer types or form a suitable tumor microenvironment promoting the growth of other cancers [10]. Regarding its role in HCC, it is not deeply explored and further research is required as reported by Yu et al. [11].

Epidemiological studies confirmed that the incidence of and mortality from HCC in females are much lower than in males suggesting that the estrogen signaling pathways may have a protective role in HCC [1]. In 2015, Wei et al. [12] suggested that 17β-estradiol (E2) and estrogen receptor (ER) signaling pathway could inhibit the progression of HCC by targeting the NLRP3 inflammasome. Although estrogen contributes to the gender disparity in HCC, to a certain degree, its precise role in HCC development remains to be investigated [13].

Therefore, the aim of the current study was to evaluate the role of NLRP3 inflammasome (-associated pyroptosis) in hepatocellular carcinogenesis by determining the gene expression status of NLRP3 inflammasome components and Gasdermin-D in the HCC tissues compared with the adjacent non-cancerous tissues. Additionally, this study aimed to clarify the potential mechanism by which E2 can be used as a protective factor (therapeutic target) against HCC.

## Subjects and methods

### Sample size calculation

The sample size was calculated using g power, referring to previous research [14] when the proportion of high NLRP3 expression in HCC cases was 0.6 and in controls 0.97 with alpha error probability 0.05 and power 80%; the calculated sample size was 19 in each group (cases and controls).

### Patients and specimens

A total number of twenty HCC cases “five females and fifteen males” with a median age of 62 years, attending the Gastroenterology Surgical Center, Faculty of Medicine, Mansoura University, from November 2019 to September 2021, were included in our study. They were diagnosed with HCC and subjected to curative surgical resection. All patients neither received radiotherapy nor chemotherapy before surgical resection. During surgery, two tissue specimens were obtained from each patient; one from the cancerous tissue just after surgical resection of HCC and the other from the corresponding adjacent non-cancerous liver tissue at least 3 cm away from the safety margin determined by the surgeon. Accordingly, these patients were classified into 2 groups as follows: “HCC group: 20 cancerous liver tissue samples and Control group: 20 adjacent non-cancerous liver tissue samples”.

All the liver tissue samples (cancerous and adjacent non-cancerous) obtained from all patients during surgery were immediately submerged in an appropriate volume of “RNA Stabilization Reagent (Qiagen, Germany, Cat. no. 76104)” with 10 μl reagent per 1 mg of tissue, incubated at 4 °C overnight, followed by total RNA extraction in the next day or storage at − 80 °C until total RNA extraction was done.

Tumor grading based on hepatic cell differentiation was determined according to the Edmondson and Steiner (ES) grading system [15, 16] and HCC staging was classified according to the TNM staging system for HCC released by the American Joint Committee on Cancer (AJCC) (8th edition) [17]. Detailed demographic and clinicopathologic characteristics of these HCC patients are described in (Table 1).

**Table 1:** Demographic and clinicopathologic characteristics of all studied HCC patients

Characteristic	*N* (%) of HCC patients
^*^Age (years) Median (25th -75th percentiles)	62 (57–66)
Sex	
Female	5 (25%)
Male	15 (75%)
History of Hepatitis C and B virus	
Positive HCV	18 (90%)
Positive HBV	0 (0%)
History of Liver Cirrhosis	17 (85%)
History of Smoking	
Current smoker	4 (20%)
Non-smoker	16 (80%)
History of Diabetes Mellitus	5 (25%)
Histopathologic type	
Hepatocellular carcinoma	19 (95%)
Fibrolamellar carcinoma	1 (5%)
Edmondson-Steiner Grading	
Grade I	2 (10%)
Grade II	14 (70%)
Grade III	4 (20%)
AJCC Staging	
Stage I	6 (30%)
Stage II	10 (50%)
Stage III	2 (10%)
Stage IV	2 (10%)
Primary tumor	
T1	7 (35%)
T2 T4	10 (50%) 3 (15%)
Lymph node involvement:	2 (10%)
Vascular invasion	13 (65%)

^*^Age is represented as median (IQR). Otherwise, data are expressed as *N* (%)

### Cell culture, reagents, and cell viability assay

The human HCC HepG2 cell line [HEPG2] was purchased from the American Type Culture Collection (ATCC^®^ HB8065^™^). The basal growth medium “DMEM/F-12” was prepared by mixing Dulbecco’s Modified Eagle Medium (DMEM) (Corning, USA) with Ham’s F-12 (Lonza Verviers SPRL, Belgium) (1:1). Then, the complete growth medium was prepared by adding 10% fetal bovine serum (FBS) (Thermo-Fisher Scientific, USA) as well as 1% penicillin–streptomycin (Lonza) to the basal growth medium. 17β-estradiol (E2) was purchased from Sigma Aldrich (Sigma Aldrich, USA, Cat.No. E8875) and 13.619 mg of E2 was dissolved in 2.5 ml of absolute ethanol to make a stock solution (20 mM). The stock solution was further diluted with a complete growth medium to yield different E2 concentrations. Vybrant MTT “3-(4, 5-dimethyl-2-thiazolyl) -2, 5-diphenyl -2H- tetrazolium bromide” Cell Proliferation Assay Kit was purchased from Thermo-Fisher Scientific (Thermo-Fisher Scientific, USA, Cat.No.V-13154).

HepG2 cells were kept in a flask containing the complete growth medium “DMEM/F-12 supplemented with 10% FBS and 1% penicillin–streptomycin” at 37 °C and 5% CO_2_ in a suitable incubator to promote attachment. When the confluence of cells reached 70–90%, the cells (5000 cell/well) were seeded into 96-well cell culture plates (3 wells/each group), allowed to adhere for 24 h, and subsequently treated with complete growth media containing different doses of E2 (0.02, 0.04, 0.06, 0.08, 0.1, 0.2, 0.4, 0.6, 0.8, 1, 10, 25, 50, 75, and 100 µM) to determine the cell viability and the half-maximum inhibitory concentration (IC50) values.

After 24, 48 and 72 h of the treatment, the effect of E2 was assessed by MTT assay according to the protocol of Roehm et al. [18]. At first, HepG2 cells were washed with phosphate-buffered saline (PBS) (Corning, USA). Then, 100 µl of a fresh complete growth medium and 10 µl of MTT (0.5 mg/ml) were added to each well including control (wells containing cells without E2 treatment) and blank wells (wells without cells), and finally, after 4 h of incubation, dimethyl sulfoxide (DMSO) was added to each well to dissolve the formazan crystals and the absorbance was read at 570 nm using an ELISA reader. All experiments were repeated three times and every time was carried out in triplicate for consistency of the result. IC50 values were calculated using 10, 25, 50, 75, and 100 µM E2 concentrations, at which a proper inhibition of the cell growth was determined, and they were 48.11 µM after 48 h of incubation and 48.02 µM after 72 h of incubation which were prepared by adding 96.2 and 96 µl of stock solution to 39903.8 and 39904 µl of complete growth medium, respectively.

### Cell culture and E2 treatment

HepG2 cells were added in 25cm^2^ flasks and divided into 3 groups (3 flasks for each group) according to E2 treatment as follows; untreated group (cells without treatment), E2-treated group after 48 h (cells treated for 48 h with 48.11 µM of E2), E2-treated group after 72 h (cells treated for 72 h with 48.02 µM of E2). After 48 h, the cells of groups 1 and 2 were harvested, while cells of group 3 were harvested after 72 h, and then RNA extraction steps were performed.

### mRNA assessment by real-time quantitative reverse transcription-polymerase chain reaction (RT-qPCR)

The NLRP3, CASP1, and GSDMD mRNA expression levels were assessed in all collected cancerous and non-cancerous liver tissue samples as well as untreated and treated HepG2 cells by total RNA extraction followed by RT-qPCR in the Medical Biochemistry Department, Faculty of Medicine, Mansoura University. Using QIAzol Lysis Reagent (Qiagen, Germany, Catalog. no. 79306), the total cellular RNA was extracted from all liver tissue samples and HepG2 HCC cells after homogenization by liquid nitrogen. The NanoDrop 2000c Spectrophotometer (Thermo Scientific, USA) was used to quantify the RNA concentration and optical density (OD) ratios. The extracted RNA samples, electrophoresed through an agarose gel, were of good quality, as indicated by a 28S:18S ribosomal RNA ratio of 2:1 and of high purity, as indicated by two OD ratios (260/280 and 260/230).

**Fig. 1:**
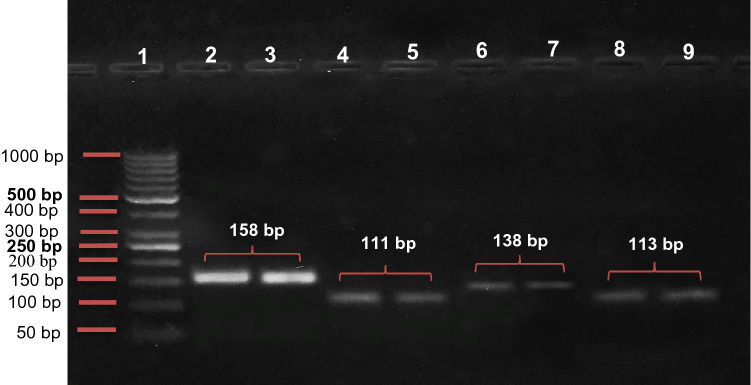
Agarose gel electrophoresis of the RT-qPCR products: **Lane 1**: 50 bp ladder, **Lane 2, 3**: GAPDH RT-qPCR products (158 bp), **Lane 4, 5**: NLRP3 RT-qPCR products (111 bp), **Lane 6, 7**: CASP1 RT-qPCR products (138 bp), **Lane 8, 9**: GSDMD RT-qPCR products (113 bp)

After that, a calculated volume of RNA (500 ng) was reverse transcribed to cDNA using the COSMO cDNA Synthesis Kit (Willowfort, UK, Cat. No.WF-10205002). Equal amounts of newly synthesized cDNA template were subjected to qPCR reaction performing on a real-time PCR instrument (Applied Biosystem 7500 PCR detection system with 96-well plates, USA), with a total reaction volume of 20 µl [10 µl of HERA ^PLUS^ SYBR Green PCR Master Mix (Willowfort, UK), 1 µl (10 pmol/µl) of gene forward primer, 1 µl (10 pmol/µl) of gene reverse primer, 6 µl of nuclease-free water and 2 µl of cDNA template]. The thermal cycler was previously programmed according to the following program; an initial denaturation at 95 °C for 2 min, followed by 40 cycles of 95 °C for 10 s and 60 °C for 30 s. The genetic sequences of the applied primer sets are provided in (Table 2) and glyceraldehyde-3-phosphate dehydrogenase (GAPDH) was used as a housekeeping gene. These primer sets were designed by Primer3 Input (version.0.4.0*) *[http://frodo.wi.mit.edu/]. Primer specificity was determined using the Primer-BLAST program, NCBI primer designing tool, (https://www.ncbi.nlm.nih.gov/tools/primer-blast/). Primer sets were prepared by Vivantis (Vivantis Technologies, Selangor Darul Ehsan, Malaysia). To confirm the specificity of the RT-qPCR products, melting curve analysis was performed. Then, the RT-qPCR products were analyzed by 3% agarose gel electrophoresis, stained with ethidium bromide, visualized by a UV transilluminator, and photographed (Fig. 1). Relative mRNA expression levels were calculated according to the **2**^**−ΔΔCt**^ method which was described by Livak and Schmittgen [19].

**Table 2:** The genetic sequences of the primer sets used in qPCR

Gene	Primer sequence	Reference Sequence	PCR product size (bp)
GAPDH	F: 5′- GTCAAGGCTGAGAACGGGAA -3′ R: 5′- AAATGAGCCCCAGCCTTCTC -3′	NM_002046.7	158
NLRP3	F: 5′- ACCTGGGGTTGTCTGAAATG -3′ R: 5′- ACTCCTACCAAGAAGGCTCAAA -3′	NM_001243133.2	111
CASP1	F: 5′- ATGAATGTCTGTGGGCAGGA -3′ R: 5′- TCAGTGTGGGAGAAAAATGAGG -3′	NM_033292.4	138
GSDMD	F: 5′- GTTCGGGGTGATGATTGAAGA -3′ R: 5′- TCTGGTGGTGTGTGCGTTG -3′	NM_1166237.1	113

### Statistical analysis

Data were entered and analyzed using IBM-SPSS software (Version 26.0. Armonk, NY: IBM Corp). Initially, quantitative data were tested for normality using Shapiro–Wilk’s test, being normally distributed if *P* > 0.050. The presence of significant outliers was tested for by inspecting boxplots. Quantitative data from each group were presented as mean ± standard deviation (SD) as data were normally distributed. “Independent-samples *t*-test” was used to compare normally distributed quantitative data between two groups, while the “One-Way ANOVA test” was used to compare normally distributed quantitative data between more than two groups. Statistically significant results were followed by post-hoc Tukey-HSD and Games-Howell tests to detect where that significant difference existed. Post-hoc Games-Howell test was used when the homogeneity of variances was violated (*P* value for Levene’s test < 0.05). The quantitative data that were not normally distributed were expressed as median and interquartile range (IQR). Spearman’s, Pearson’s, and Point-biserial correlation tests were used to determine associations between variables. For any used test, the results were considered as statistically significant if the p value is ≤ 0.05.

## Results

### The mRNA expression levels of the NLRP3 inflammasome components (NLRP3 and CASP1) and of the executioner of pyroptosis (GSDMD) in HCC tissues in comparison to adjacent non-cancerous tissues

**Fig. 2:**
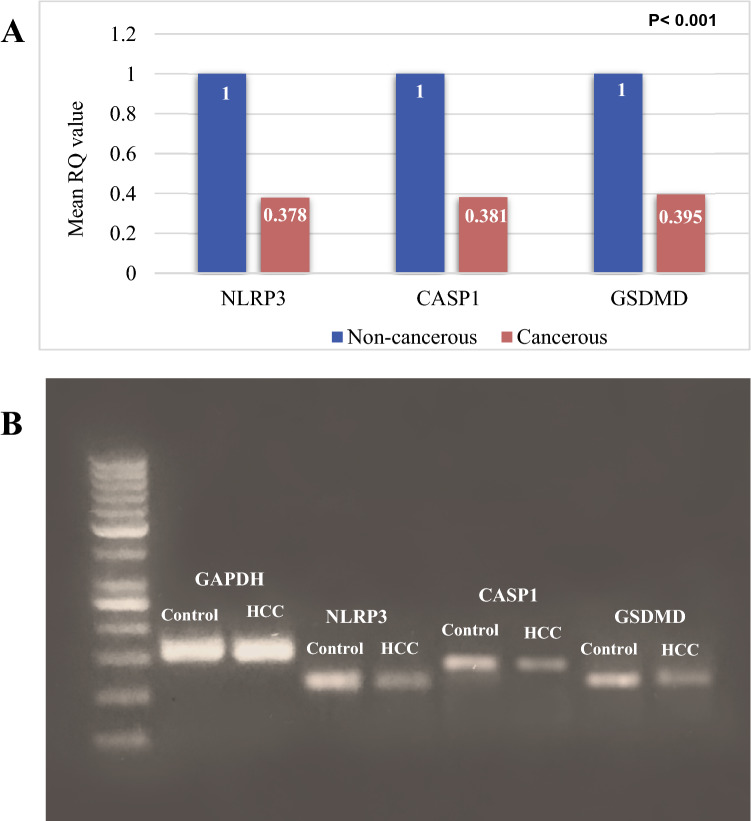
The mRNA expression levels of NLRP3, CASP1, and GSDMD in HCC patients. **A** The mRNA expression levels of NLRP3, CASP1, and GSDMD were evaluated by RT-qPCR in the cancerous as well as non-cancerous liver tissues obtained from all HCC patients. **B** Agarose gel electrophoresis of RT-qPCR products showing variations in the NLRP3, CASP1, and GSDMD mRNA expression levels in the paired cancerous and non-cancerous liver tissues obtained from one representative case. "Data are expressed as mean ± SD. Test of significance is Independent-samples *t*-test; p value is considered significant when it is ≤ 0.05"

The mRNA expression levels of NLRP3, CASP1, and GSDMD were evaluated in 20 sample pairs of HCC tissues and adjacent non-cancerous liver tissues showing a statistically significant difference between them in the mRNA expression levels of these three genes being under-expressed in HCC tissues (*P* < 0.001) (Fig. 2A, B).

### NLRP3, CASP1, and GSDMD mRNA expression levels in different HCC grades and stages in comparison to control

**Fig. 3:**
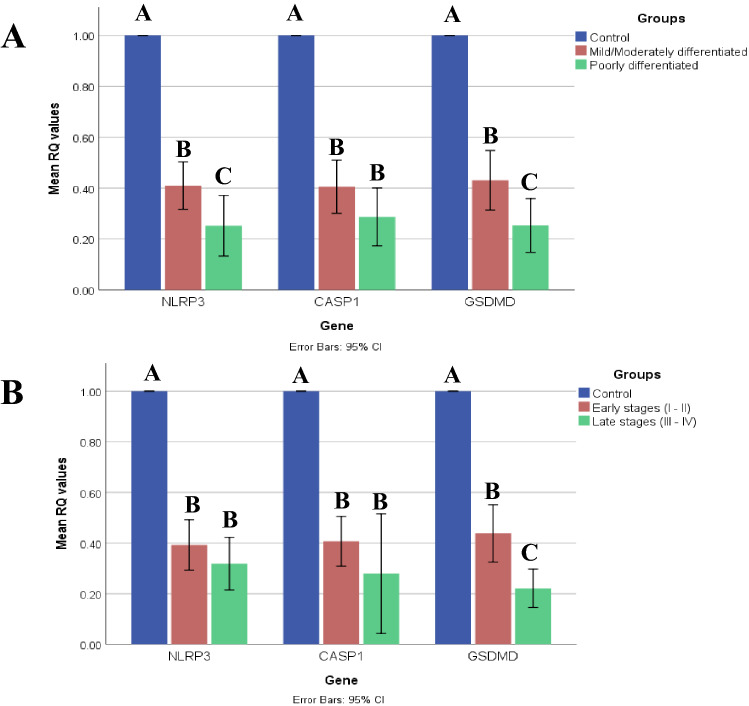
NLRP3, CASP1, and GSDMD mRNA expression levels in different HCC grades and stages. **A** NLRP3, CASP1, and GSDMD mRNA expression levels in mild/moderately differentiated HCC and poorly differentiated HCC in comparison to control. **B** NLRP3, CASP1, and GSDMD mRNA expression levels in early and late HCC stages in comparison to the control group. "Data are expressed as mean ± SD. Test of significance is One-Way ANOVA test; post-hoc test, pairwise comparison using Games-Howell adjustment is presented as capital letters (similar letters = statistically insignificant difference, different letters = statistically significant difference), p value is considered significant when it is ≤ 0.05"

As illustrated in (Fig. 3), NLRP3, CASP1, and GSDMD mRNA expression levels were statistically significantly lower in different HCC grades (mild/moderately differentiated HCC “grade I-II” and poorly differentiated HCC “grade III”) and stages (early and late) than those levels observed in the control group (*P* < 0.001). Post-hoc Games-Howell test revealed that grade III HCC showed lower mRNA expression levels than grades I-II which were statistically significant for NLRP3 (*P* = 0.043) and GSDMD (*P* = 0.035), but not for CASP1 (*P* = 0.159) (Fig. 3A). The same test also indicated lower mRNA expression levels in the late stages than those levels in the early stages which were statistically significant for GSDMD (*P* = 0.004), but not for NLRP3 (*P* = 0.416) and CASP1 (*P* = 0.377) (Fig. 3B).

### Correlations between the mRNA levels of NLRP3, CASP1, and GSDMD in HCC tissues and clinicopathologic as well as demographic characteristics of the studied HCC patients

As shown in (Table 3), there were statistically significant negative correlations between mRNA expression levels of NLRP3 and GSDMD with different HCC grades (P values were 0.008, 0.027, respectively). For CASP1, there was a negative correlation of medium strength between its mRNA expression levels and different HCC grades, but it didn’t achieve a statistical significance (coefficient > 0.3, but *P* > 0.05). Regarding AJCC staging, mRNA expression levels of CASP1 and GSDMD had negative correlations of medium strength with different stages of HCC, but they didn’t achieve a statistical significance (*P* > 0.05). Otherwise, the mRNA levels of these three genes did not have any statistically significant negative or positive correlation with other clinicopathologic characteristics, age, or sex in all studied HCC patients (*P* > 0.05).

**Table 3:** Correlations between NLRP3, CASP1, and GSDMD expression levels in HCC tissues and clinicopathologic as well as demographic characteristics

Parameter	NLRP3		CASP1		GSDMD	
	Coefficient	P value	Coefficient	*P* value	Coefficient	*P* value
ES Grading	− 0.575	**0.008**	− 0.375	0.103	− 0.493	**0.027**
AJCC Staging	− 0.072	0.763	− 0.354	0.125	− 0.336	0.147
^*^Liver cirrhosis	− 0.311	0.182	− 0.096	0.686	− 0.074	0.756
Primary tumor	− 0.101	0.670	− 0.145	0.541	− 0.212	0.370
^*^LN involvement	− 0.021	0.931	− 0.086	0.719	− 0.299	0.200
^*^Vascular invasion	− 0.117	0.623	− 0.159	0.503	− 0.189	0.425
Age	− 0.171	0.472	− 0.320	0.169	− 0.267	0.254
^*^Sex	− 0.166	0.486	− 0.271	0.249	− 0.249	0.291

Tests of significance are Spearman’s correlation test and *Point-biserial correlation test

*P* value is considered significant if it is ≤ 0.05

### Correlations between the NLRP3, CASP1, and GSDMD mRNA expression levels in all studied HCC patients

**Fig. 4:**
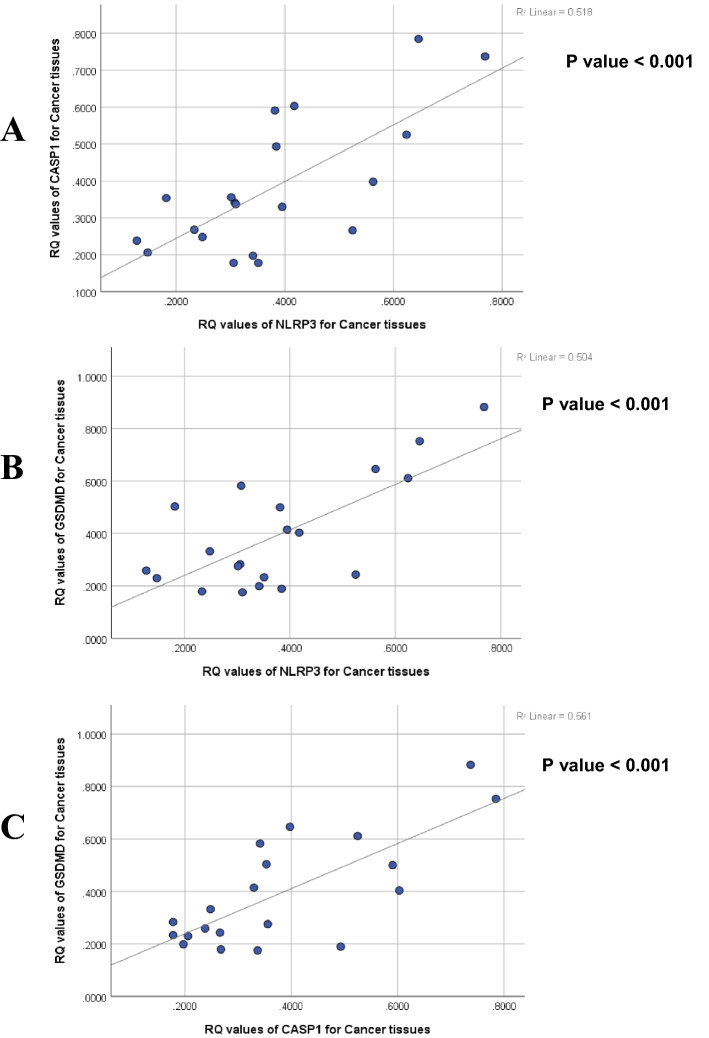
Correlations between NLRP3, CASP1 and GSDMD genes in all studied HCC patients. **A** Correlation between NLRP3 and CASP1 mRNA levels. **B** Correlation between NLRP3 and GSDMD mRNA levels. **C** Correlation between CASP1 and GSDMD mRNA levels. "Test of significance is Pearson’s correlation test; p value is considered significant when it is ≤ 0.05"

Statistical analysis of the RQ values of NLRP3, CASP1, and GSDMD showed that mRNA expression levels of the three studied genes had statistically significant strong positive correlations with each other (*P* < 0.001) (Fig. 4A–C).

### Effect of 17β-estradiol treatment on the HepG2 HCC cell line

**Fig. 5:**
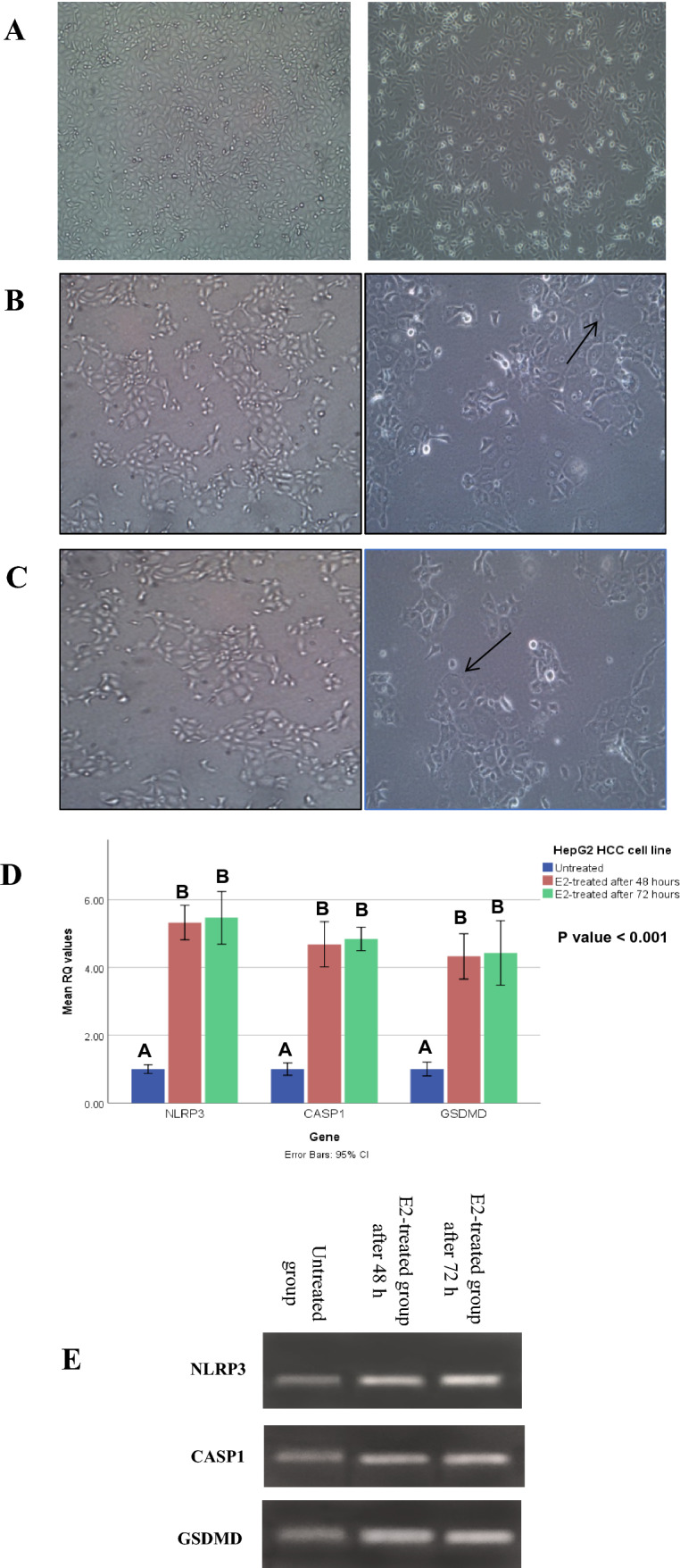
17β-estradiol can reverse the malignant behavior of the HepG2 HCC cell line by overexpressing the NLRP3 inflammasome components and the executioner of pyroptosis. (**A**) HepG2 HCC cells without treatment (control group). **B** and **C** Microscopic evidence of decreased cell number and morphological changes of HepG2 HCC cells treated with: **B** 48.11 µM of E2 after 48 h and **C** 48.02 µM of E2 after 72 h. **D** NLRP3, CASP1, and GSDMD expression levels in the E2-treated groups compared with the untreated group **E** Agarose gel electrophoresis of RT-qPCR products showing variations in the NLRP3, CASP1, and GSDMD mRNA expression levels in the untreated group, E2-treated group after 48 h and E2-treated group after 72 h. "Data are expressed as mean ± SD. Test of significance is One-Way ANOVA test; post-hoc test, pairwise comparison using Tukey-HSD adjustment is presented as capital letters (similar letters = statistically insignificant difference, different letters = statistically significant difference), p value is considered significant when it is ≤ 0.05"

The HepG2 HCC cells were treated with E2 (48.11 µM for 48 h and 48.02 µM for 72 h), then they were examined by an inverted microscope showing that E2 can inhibit cell proliferation and also cause morphological changes in E2-treated HepG2 HCC cells (Fig. 5A–C).

### Effect of 17β-estradiol treatment on the expression of the three studied genes in HepG2 HCC cell line by RT-qPCR

Real-time PCR analysis revealed that NLRP3, CASP1, and GSDMD mRNA expression levels were significantly increased in E2-treated HepG2 HCC cells. As described in (Fig. 5D, E), the mRNA expression of NLRP3, CASP1, and GSDMD was statistically significantly different between the three groups (untreated group, E2-treated group after 48 h, and E2-treated group after 72 h) (*P* < 0.001). Post-hoc Tukey-HSD tests showed that there was a statistically significantly higher gene expression in each of the two E2-treated groups vs. the untreated group, but not between the two E2-treated groups.

## Discussion

HCC is the sixth most common cancer all over the world [20]. Several cellular and molecular mechanisms are involved in the pathogenesis of HCC including necroinflammation, hypoxia, oxidative stress, mitochondrial damage, changes in the tumor microenvironment, activation of cytokines and growth factors, and genetic alterations [21]. The emergence of immune- and genomic- based therapies has been transformed the treatment strategy of many types of cancer and these therapies are starting to be applied to HCC [22]. Therefore, a deep exploration of these different mechanisms involved in HCC is urgently needed to find effective biomarkers and specific therapeutic targets to improve the treatment efficacy [23].

One key step in the inflammatory response, generated by innate immunity to protect the liver against various pathogens and danger signals, is the activation of inflammasomes. The NLRP3 inflammasome has a double-edged sword effect in cancer which is dependent on many factors such as the expression of its components, the presence of mutations that affect its expression, cancer type, and stages of tumorigenesis [24]. However, its direct role in HCC remains poorly described as reported by García-Pras et al. [8].

The present study involved 20 patients with different grades and stages of HCC. Seventeen of these patients developed HCC in a background of liver cirrhosis. The NLRP3, CASP1, and GSDMD expression levels were evaluated in HCC tissues and adjacent non-cancerous liver tissues obtained from these HCC patients aiming to clarify their role in the development and progression of HCC. Our results showed a statistically significant under-expression of NLRP3 and CASP1 genes in HCC tissues when compared to the adjacent non-cancerous tissues.

These results correspond with the data of Wei et al. who observed that the NLRP3 inflammasome components (NLRP3, ASC, and CASP1) were significantly decreased at both mRNA and protein levels in the HCC tissues in comparison with the adjacent non-cancerous liver tissues suggesting that lack of this multi-protein complex was involved in HCC development [14].

Fujikawa et al. firstly reported that caspase-1 protein levels were significantly lower in the HCC tissues than those levels in the adjacent non-cancerous tissues indicating its association with HCC development [25]. Moreover, Chu et al. assessed both protein and mRNA expression levels of CASP1 in Human HCC tissues and in HCC cell lines using Western blot and RT-qPCR. They found that CASP1 levels were significantly lower in HCC tissues and HCC cell lines than those levels in adjacent non-cancerous tissues and normal hepatocyte cell lines suggesting that loss of CASP1expression is involved in the pathogenesis of HCC [26].

Inflammation was defined as one of the hallmarks of tumorigenesis. In its early phases, the immune system can suppress it via NLRP3 inflammasome activation and IL-18 secretion which in turn can trigger the NK cells to exert their cytotoxic potential against cancer cells. Nevertheless, continuous unprovoked activation of this inflammasome and presence of IL-1β in the tumor microenvironment can attract immunosuppressive cells including tumor-associated macrophages (TAMs), myeloid-derived suppressor cells (MDSCs) as well as regulatory T cells (Tregs) which promote tumor invasion and metastasis [27]. HCC typically originates on the top of chronic hepatic inflammation which is almost accompanied by a massive loss of hepatocytes and irreversible liver damage. In turn, dying hepatocytes release DAMPs which aggravate inflammation and then induce further liver damage generating a highly hepatotoxic cycle of inflammation, cell death, and compensatory liver regeneration increasing the risk of liver cancer. Thus, hepatocytes proliferating under conditions of continuous liver injury may accumulate mutations leading to dysplasia and finally to tumor development [28]. Once HCC formed, the NLRP3 inflammasome components were significantly under-expressed in the transformed liver cancer cells demonstrating that these malignant cells originated from NLRP3 inflammasome-deficient precursor cells [14].

Parallel previous findings in colorectal cancer mice models [29–32], multiple myeloma patients [33], and lung cancer patients [34] suggesting a protective role of the NLRP3 inflammasome against cancer development as they showed that NLRP3 and/or CASP1 levels were significantly downregulated in these different cancers.

In contrast, several studies suggested that NLRP3 inflammasome components were overexpressed in many types of cancer such as gastric cancer, endometrial carcinoma, laryngeal squamous cell carcinoma and colorectal cancer demonstrating that NLRP3 inflammasome activation may contribute to the development and progression of these different cancers [35–38]. All these reported data suggest that a united mechanism for activating of NLRP3 inflammasome and its contribution to cancer has not emerged yet, so further studies are needed to clarify its role in each kind of tumor.

In the present study, NLRP3 and CASP1 expression levels are lower in poorly differentiated HCC (grade III) when compared with mild/moderately differentiated HCC (grade I-II) which are statistically significant for NLRP3, but not for CASP1. Also, there is a statistically insignificant decrease in NLRP3 and CASP1 expression levels in late HCC stages when compared with early ones suggesting that the HCC patients with lower NLRP3 and CASP1 expression levels are prone to have more advanced stages (stages III and IV) and poorer cancer cell differentiation (Grade III). These findings are in line with Wei et al. who demonstrated that the patients with advanced HCC stage were likely to have lower expression levels of the NLRP3 inflammasome components, whereas HCC patients with higher grade had weaker immunoreactivity to these components [14].

Gasdermin-D has a pore-forming activity and is well-recognized as an executor for pyroptosis [39]. However, the role of GSDMD itself in HCC development and progression remains unclear. Hence, the present study also analyzed the mRNA expression levels of GSDMD showing that its expression levels were statistically significant under-expressed in HCC tissues suggesting that deficiency of GSDMD may be involved in HCC development. Similarly, other previous studies revealed that GSDMD expression was significantly downregulated in other types of cancer such as gastric cancer and colorectal cancer [40, 41].

In contrast, Lv et al. found that HCC tissues and metastatic HCC tissues exhibited overexpression of GSDMD mRNA level when compared with normal and non-cancerous tissues hypothesizing that GSDMD might induce HCC progression. They also found that “the high mobility group box 1/toll-like receptor 4/caspase-1 activation pathway” may contribute to GSDMD over-expression and its cleavage which then can promote HCC tumorigenesis [42]. Similar studies also demonstrated that GSDMD expression was significantly upregulated in other cancers such as lung cancer and glioma [43, 44].

In the current study, statistically significant lower GSDMD mRNA levels are noticed in grade III HCC when compared with grades I-II as well as in late HCC stages when compared with early ones. These findings suggest that GSDMD under-expression is closely related to late HCC stages and poorly differentiated carcinoma.

According to the current study, a correlation analysis was performed showing that NLRP3 mRNA expression had a statistically significant negative correlation with HCC grades, but not with stages of HCC. Also, CASP1 mRNA expression levels were negatively correlated with different grades and stages of HCC but without a statistical significance. In addition, there was a statistically significant negative correlation between the mRNA expression levels of GSDMD and different HCC grades. Also, there was a negative correlation between the mRNA expression levels of GSDMD and HCC staging but without a statistically significance. These findings suggested that the lower the mRNA expression level, the higher the grade and the later the stage of HCC.

These results are in agreement with Wei’s findings which revealed that the expression of NLRP3 inflammasome components was correlated inversely with the pathological grades as well as advanced stages of HCC indicating that loss of NLRP3 inflammasome was involved in HCC progression [14].

Our data also reveals that NLRP3, CASP1 and GSDMD mRNA expression levels in HCC patients had statistically significant strong positive correlations with each other. Also, Wei et al. observed that the NLRP3, CASP1, and ASC expression levels had significantly positive correlations with each other suggesting that these components cooperated to contribute to HCC development [14]. Pyroptosis is dependent on caspase-1 activation and gasdermin cleavage to induce cancer cell death. It is involved in the pathologic process of various kinds of cancers and its beneficial effect against HCC was reported by Chu et al. [26]. The present study provided evidence for the involvement of NLRP3 inflammasome and pyroptosis in HCC as revealed by the statistically significant under-expression of NLRP3 (the sensor of inflammasome), CASP1 (the effector of inflammasome), and GSDMD (the downstream molecule of NLRP3 inflammasome activation and the executioner of pyroptotic cell death) in the cancerous tissues obtained from HCC patients.

Similarly, Zhang et al. found that CASP1 is downregulated in HCC tissues when compared to the normal liver tissues indicating that the canonical pyroptotic pathway is possibly repressed in the pathogenesis of HCC [45]. On the other hand, inhibition of the pyroptotic signaling pathways has been reported to possess anti-HCC effects. Fan et al. showed that the proliferation, invasion, and metastasis of HCC cells are suppressed through NLRP3 inflammasome inhibition [46]. This difference is probably related to the degree of activation of pyroptosis. Minor activation of pyroptosis induces a minority production of IL-1β which promotes the proliferation of liver cancer, while severe activation of this pathway causes the death of HCC cells [47].

Epidemiological studies have revealed that females have a lower incidence and reduced mortality from HCC than males suggesting a protective role of the estrogen signaling against liver cancer [1]. In 2019, Wei et al. found that 17β-estradiol exerted anticancer effects in HCC by targeting the NLRP3 inflammasome however, its precise role in HCC development remains to be investigated [13].

So in the present work, we treated the HepG2 HCC cell line with 17β-estradiol for 48 and 72 h. Then, the NLRP3, CASP1, and GSDMD mRNA expression levels were analyzed to define if there is a potential link between the NLRP3 inflammasome and estrogen. Also, to clarify the potential molecular mechanism by which E2 can be used as a protective factor against HCC development.

By inverted microscope, our results revealed that E2 inhibits cell proliferation and causes morphological changes in the treated HepG2 HCC cells. Also, we found that E2 can activate NLRP3 inflammasome and induce pyroptosis in HCC cells as demonstrated by the statistically significantly higher mRNA expression levels of NLRP3, CASP1, and GSDMD in each of the two treated groups when compared with the untreated group. All these findings indicate a protecting role of estrogen against HCC development and its progression.

In partial agreement with these findings, Wei et al. demonstrated a significant positive correlation between the expression level of NLRP3 inflammasome components and estrogen receptors beta (ERβ). They also showed that after treatment with E2, proliferation, migration, and colony formation capabilities of HCC cells were significantly inhibited in an ERβ/MAPK/ERK pathway-dependent manner which is consistent with a significantly upregulated expression and activation of NLRP3 inflammasome [12]. Mitogen-activated protein kinase pathway is one of the signaling pathways that regulate the NLRP3inflammasome activation [48]. MAPKs mainly include p38, extracellular signal-regulated kinase (ERK1/2), and several c-Jun N terminal kinases (JNKs) [49]. Phosphorylation of NLRP3 at the Ser198 residue by JNK1 is critical for its deubiquitination facilitating its self-association as well as subsequent inflammasome complex assembly and activation [50].

Also, Wei et al. showed that E2-induced activation of the NLRP3 inflammasome promoted caspase-1-dependent pyroptotic cell death in HCC cells which may be a potential neoplastic target for the treatment of HCC [13]. In addition, Zhang et al. revealed a tumor-suppressor function of the NLRP3 inflammasome activation in HCC represented by induction of pyroptosis indicated by the elevated expression levels of NLRP3, cleaved caspase-1, and also an increased level of GSDMD-NT domain [51].

The protecting role of 17β-estradiol in HCC has been attributed to the anti-proliferative and anti-inflammatory activities brought by E2 through binding to and activation of ERβ [52, 53]. Initiation of both genomic and non-genomic estrogen signaling pathways depends on E2 binding to its receptor. In the genomic pathway, E2 binds to nuclear ERs activating them which in turn stimulate the expression of target genes either by direct binding to specific DNA sequences called estrogen response elements (EREs) or tethering where ER interacts with DNA-bound transcription factor in a way that stabilizes the DNA binding of that transcription factor resulting in enhanced transcription of target genes [54]. In the non-genomic pathway, E2 binding to membrane-associated ERs may exert rapid actions which start with the activation of a variety of signal transduction pathways such as MAPK/p38, MAPK/ERK, PLC/PKC, and PI3K/AKT leading to regulation of gene expression and cytoplasmic alterations [55].

Regarding all these data, we suggest that after treating HepG2 HCC cells with 17β-estradiol, it binds to and activates ERs which in turn can upregulate the expression of NLRP3 inflammasome components and also can activate the MAPK signaling pathway resulting in phosphorylation of NLRP3 protein. This phosphorylation may be responsible for the NLRP3 inflammasome activation. Once it becomes activated, active caspase-1 can trigger the pyroptotic cell death via cleavage of gasdermin-D which perforates the cell membrane by its amino-terminal domain [56].

In conclusion, we found that in combination with each other, NLRP3, CASP1, and GSDMD possibly serve as promising therapeutic targets for HCC based on the under-expression of the NLRP3 inflammasome components (NLRP3 and CASP1) and the executioner of pyroptosis (GSDMD) in the studied HCC patients. In addition, our study suggests that 17β-estradiol may be effective in HCC treatment as it can inhibit tumor cell growth and proliferation by activating the NLRP3 inflammasome and the caspase-1-dependent pyroptosis in HCC cells.

## Data Availability

Data are available.
